# R-spondins are involved in the ovarian differentiation in a teleost, medaka (*Oryzias latipes*)

**DOI:** 10.1186/1471-213X-12-36

**Published:** 2012-12-07

**Authors:** Linyan Zhou, Tapas Charkraborty, Xiangguo Yu, Limin Wu, Gang Liu, Sipra Mohapatra, Deshou Wang, Yoshitaka Nagahama

**Affiliations:** 1 Key Laboratory of Freshwater Fish Reproduction and Development (Ministry of Education), School of Life Science, Southwest University, Chongqing, 400715, P.R. China; 2 Division of Molecular Environmental Endocrinology, National Institute for Basic Biology, Okazaki, 444-8585, Japan; 3 Japan Society for the Promotion of Science (JSPS), 8 Ichiban-cho, Chiyoda-ku, Tokyo, 102-8472, Japan; 4 Institution for Collaborative Relations, Ehime University, 3 Bunkyo-cho, Matsuyama, 790-8577, Japan

**Keywords:** *Rspo1, 2, 3*, Molecular cloning, Expression profiles, Ovarian differentiation

## Abstract

**Background:**

In mammals, R-spondin (Rspo), an activator of the Wnt/β-catenin signaling pathway, has been shown to be involved in ovarian differentiation. However, the role of the Rspo/Wnt/β-catenin signaling pathway in fish gonads is still unknown.

**Results:**

In the present study, full-length cDNAs of *Rspo1, 2* and *3* were cloned from the gonads of medaka (*Oryzias latipes*). The deduced amino acid sequences of *mRspo1-3* were shown to have a similar structural organization. Phylogenetic analysis showed that Rspo1, 2 and 3 were specifically clustered into three distinct clads. Tissue distribution revealed that three *Rspo* genes were abundantly expressed in the brain and ovary. Real-time PCR analysis around hatching (S33-5dah) demonstrated that three *Rspo* genes were specifically enhanced in female gonads from S38. *In situ* hybridization (*ISH*) analysis demonstrated that three *Rspo* genes were expressed in the germ cell in ovary, but not in testis. Fluorescence multi-color *ISH* showed that *Rspo1* was expressed in both somatic cells and germ cells at 10dah. Exposure to ethinylestradiol (EE2) in XY individuals for one week dramatically enhanced the expression of three *Rspo* genes both at 0dah and in adulthood.

**Conclusions:**

These results suggest that the Rspo-activating signaling pathway is involved in the ovarian differentiation and maintenance in medaka.

## Background

Sex determination in mammals occurs through inheritance of the X or Y sex chromosome from the parents. In mice, the presence of the male-determining *Sry* gene directs the undifferentiated gonad to develop into a testis by promoting the expression of *Sox9* and *Fgf9*[1,2]. Early ovarian development has long been thought of as a default pathway switched on passively by the absence of *Sry* gene. Recent genetic and transcriptomic studies challenge this view and show that two master pathways simultaneously repress male-specific genes and activate female-specific genetic cascades. This antagonistic action is maintained from embryonic stages to adulthood [3]. Several reports revealed that a Foxl2-leading pathway and Rspo1-activating signaling pathway act independently and complementary to each other to promote ovarian development [4-6].

Studies suggest that all four members of the Rspo family play a key role in embryogenesis, development and tumorigenesis. The mammalian Rspo family is comprised of 4 members (Rspo1-4) with a similar domain organization and regulates the WNT signaling pathway via a common mechanism [7]. R-spondins function as ligands of the orphan receptors LGR4 and LGR5 to regulate Wnt/β-catenin signaling [8,9]. Disruption of the human *RSPO1* gene in a recessive syndrome was characterized by XX sex reversal, palmoplantar hyperkeratosis and a predisposition to squamous cell carcinoma of the skin [10]. Additionally, RSPO1 was also demonstrated as a potent and specific mitogen for the gastrointestinal epithelium, in order to promote the proliferation of intestinal crypt cells [7]. Rspo2 also appears to play an essential role in muscle development in both mouse and *Xenopus* embryos [11]. Since *Rspo2(−/−)* mice exhibited midfacial skeletal defects, lim loss and lung hypoplasia, it might be indicated that Rspo2 regulates midfacial, limb, and lung morphogenesis during development through the Wnt/β-catenin signaling [12]. Mutation of the *Rspo2* gene resulted in the formation of short hair on the head, face, and lower legs in the Portuguese water dog [13]. Knockdown of *Rspo3* in *Xenopus* embryos induces vascular defects suggesting its key role in vasculogenesis and angiogenesis. Targeted disruption of mouse *Rspo3* leads to embryonic lethality caused by vascular defects and remodeling of the vascular plexus in the placenta or impaired formation of the labyrinthine layer of the placenta [14]. Congenital mutations in *RSPO4* resulted in anonychia with the absence of all fingernails and toenails in humans, and *RSPO4* mutations preferentially clustered in the furin-like cysteine-rich domains [15,16].

Recently, much attention has been paid to the role of the Rspo1-activating signaling pathway in the reproductive system, especially in early sex determination and differentiation. In vertebrates, *Rspo1* displays a conserved, female-specific increase in expression in several species [17-19]. Investigations in mammalian species have demonstrated that RSPO1 is postulated to switch on ovarian determination and differentiation by synergizing with specific Wnt ligands to stabilize the intracellular canonical β-catenin signaling pathway, which in turn activates ovarian differentiating genes in the bipotential gonad [20]. Mutations of *RSPO1* in humans induce testis formation and male development in XX individuals, in the absence of *SRY*[10]. In the mouse, ovarian differentiation requires activation of the RSPO1/WNT/β-catenin signaling pathway in both somatic cells and germ cells. In *Rspo1*^*−/−*^ XX gonads, severe impairments i.e. germ cell proliferation, expression of the early meiotic marker *Stra8* and entry into meiosis were observed. The author proposed that RSPO1/β-catenin signaling is involved in meiosis in fetal germ cells and contributes to the cellular decision of germ cells to differentiate into oocytes or sperms [21]. In the goat gonads, both *Rspo1* and *2* showed a female-specific expressional profile from 36 day post coitus (*dpc*) to adulthood. Therefore, goat *Rspo1* was correlated with germ line cell differentiation before and during meiosis, while *Rspo2* was considered as a candidate gene for ovarian differentiation. Goat *Rspo4* was also specifically expressed in both the XX female gonad from 50 to 90 *dpc*, although only very faintly. However, *Rspo3* was equally expressed in XX and XY gonads [5]. Except for goat, the expression and potential roles of all Rspo family members in other vertebrates are largely unknown.

Medaka has been used as an ideal model to study sex determination and differentiation with XX-XY genetic system and small genome [22]. *DMY/Dmrt1b* has been identified as the male sex determining gene of medaka, which initiates the development of testes in XY males by inhibiting male primordial germ cell (PGC) proliferation at the sex-determining stage [23,24]. Conversely, it is well accepted that estrogen is essential for ovarian differentiation and maintenance in female fish [25-27]. It was well documented that estrogen is necessary for the maintenance of *Rspo1* expression in a direct or indirect manner in oviparous species [19]. Recent studies revealed that Wnt signaling is implicated in multiple processes of male and female gonadal development in rainbow trout [28]. Therefore, it is essential and critical to explore whether Rspo/Wnt/β-catenin signaling pathway plays a key role in fish sex determination and differentiation, just like its role in mammals. In this study, we investigated the temporal and spatial expression profiles of *Rspo1*, *2* and *3* in the gonads during early ontogenic stages, meanwhile their expression profiles by steroid treatment were also examined. To our knowledge, this is the first report that Rspo family members might be critical for ovarian differentiation and maintenance in fish.

## Results

### Sequence analysis

In this study, three *Rspo* genes were cloned in medaka. The ORF of *Rspo1, 2* and *3* contained 810 bp, 738 bp and 984 bp encoding 270, 246 and 328 amino acids (aa), respectively. Sequence analysis revealed that medaka Rspo1 displayed higher identity to tilapia (84%) and zebrafish (74%) than its mammalian counterpart i.e. human (62%), mouse (62%), chicken (64%). The putative amino acid sequence of medaka Rspo2 also revealed higher similarity to zebrafish (73%) than human (69%), mouse (68%), chicken (69%) and *Xenopus* (69%). However, the deduced amino acid sequence of medaka Rspo3 showed relative lower homology to zebrafish (58%), human (46%), mouse (46%), chicken (45%), and *Xenopus* (41%) Rspo3.

**Figure 1:**
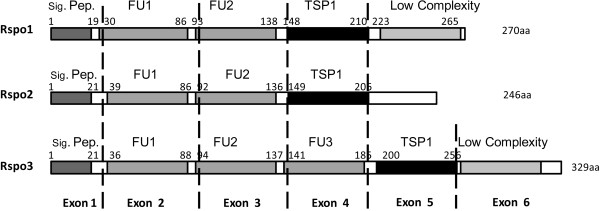
**Exon organization and conserved domain structure of three medaka R-spondins.** The medaka Rspo1 and 2 protein genes share a common organization, each consisting of five coding exons corresponding to predicted structural domains. Predicted domains include a leading signal peptide (Sig. Pep), two furin-like type Cys-rich domains (FU1, FU2), a thrombospondin-type domain (TSP) and low complexity (white color). Additionally, Rspo3 protein possesses an extra furin-like type Cys-rich domain (FU3) located in exon 4. Conserved domain residues and exon boundaries are indicated by rectangle (gray or black) and dotted lines, respectively.

Similar to mammalian species, medaka *Rspo1* and *2* contain five exons by a structural analysis. However, fish *Rspo3* from medaka and zebrafish includes 6 exons. Three fish Rspo family proteins share substantial structural homology and possess one signal peptide at the N-terminal, two or three conserved cystine-rich furin-like domains (FU) and a thromobospondin-1 domain (TSP-1) (Figure 1). The C-terminal sequences of the three Rspo proteins were found to be less conservative.

### Phylogenetic analysis

**Figure 2:**
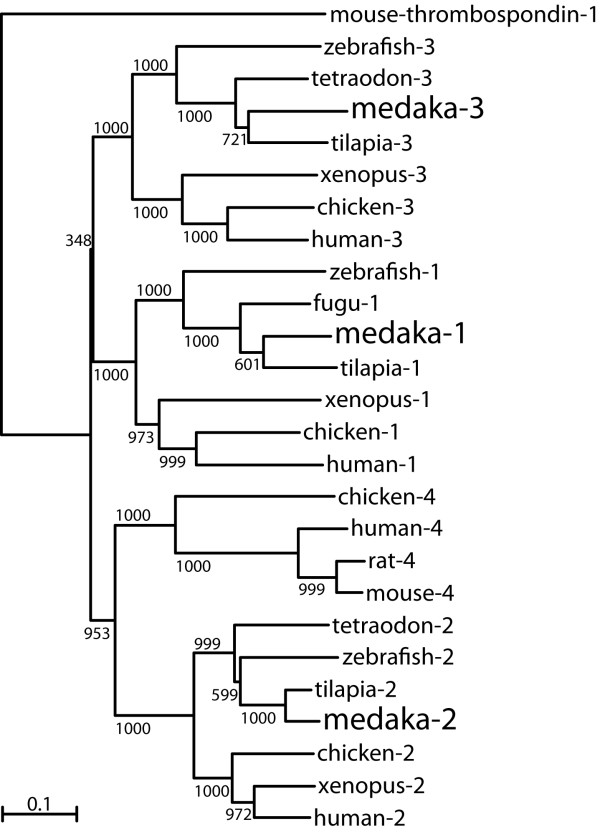
**Phylogenetic tree of Rspo family proteins of vertebrates was constructed by using mouse thrombospondin-1 as an outgroup.** Values on the tree represent bootstrap scores of 1000 trials, indicating the credibility of each branch. Branch lengths are proportional to the number of amino acid changes on the branch. Refer to *Materials and Methods* for GenBank accession nos.

To understand the phylogenetic relationship of RSPO family members among vertebrates, a phylogenetic tree was constructed based on the amino acid sequences of Rspo1, 2, 3 and 4 from different species (Figure 2). Phylogenetic analysis demonstrated that Rspo1, 2 and 3 from medaka along with their mammalian counterparts were clustered into three distinct clads. However, Rspo4 couldn’t be isolated from the available genome DNA databases in fish.

### Tissue distribution

**Figure 3:**
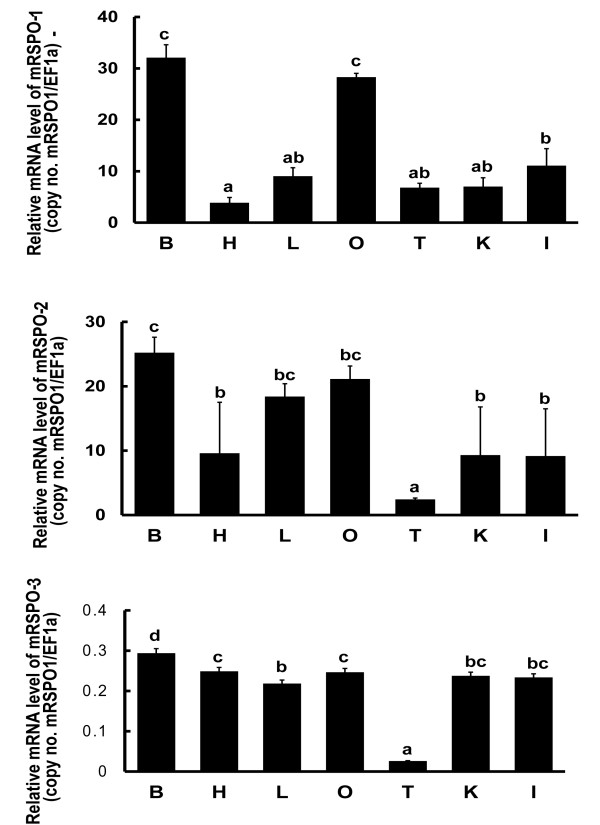
**Real-time PCR analysis of *Rspo1*, *2 *and *3 *in various tissues of adult medaka.** B, brain; H, heart; L, liver; O, ovary; T, testis; K, kidney; I, intestine. Relative mRNA level (copy number of each gene/*EF1*-α) represents the mean ± S.E. of samples from at least 3 different fish. Values with different letters indicate significant difference (P < 0.05) of each gene in different tissues.

Various tissues were collected from adult medaka for RNA extraction and cDNA synthesis which was used as templates for real time PCR analysis. The tissue distribution analysis revealed that *Rspo1* and *2* were ubiquitously expressed in the brain, liver, heart, intestine, kidney, ovary and testis, with dominant expression in the brain, liver and ovary. *Rspo3* was expressed at almost the same level in all the checked tissues except testis. The mRNA levels of *Rspo1* and *2* were much higher than that of *Rspo3* in gonads. Importantly, sexually dimorphic expression profiles of these genes were found in the gonads with much higher levels in the ovary than in the testis (Figure 3).

### Ontogenic expression of *Rspo1, 2* and *3* by real-time PCR

**Figure 4:**
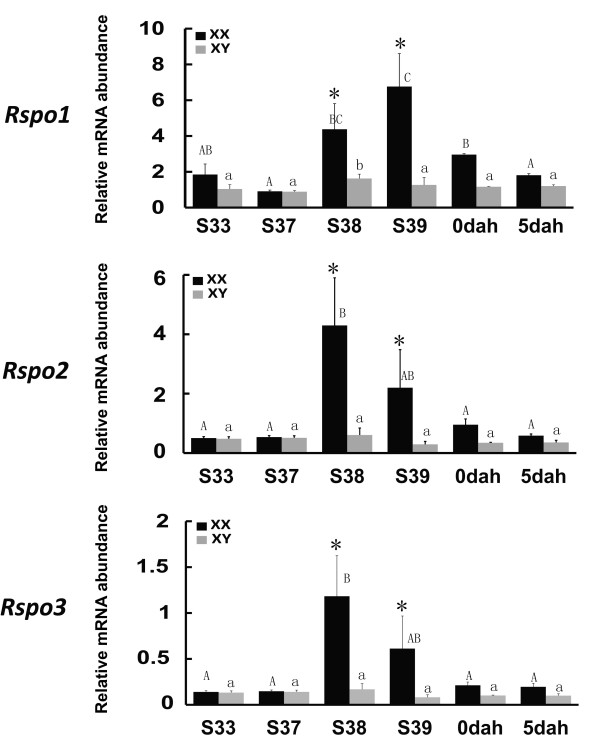
**Expression profiles of *Rspo1,2 *and *3 *during the critical period of sex determination/differentiation in the medaka gonads assayed by real-time PCR.** Relative mRNA level (copy number of each gene/*EF1*-α) represents the mean ± S.E. of samples obtained from at least 15 fish at each developmental stages. Mean values with different uppercase, different lowercase and asterisks indicate significant difference of each gene in the female gonads, male gonads, between female and male gonads, respectively (P < 0.05). (▮ = female □ = male; S = stage; dah = day after hatching).

In medaka, the first morphological sex difference manifested in the gonads reflects that the female-specific germ cell proliferation starts from stage 38 before hatching [29]. A real-time PCR analysis was carried out to investigate the expression profiles of three *Rspo* genes during the critical period of sex determination/differentiation (Figure 4). Intriguingly, female-specific increase in *Rspo1*, *2* and *3* expression profiles were detected at S38 and S39 when the first morphological sexual differentiation occurs in medaka, while it was decreased from 0dah. In contrast, in males, the expression of all three *Rspo* genes remained at a much lower level during those stages.

### Expression of *Rspo1, 2* and *3* in the gonads by *ISH*

**Figure 5:**
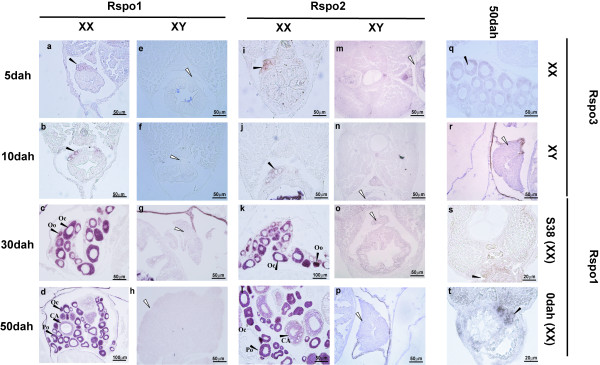
**Expression of *Rspo1,2 *and *3 *in the gonads of medaka analyzed by *ISH.****Rspo1* and *2* were expressed in both the germ cell and somatic cell in the XX gonads at the early stages (S38 to 10dah) (*Rspo1*: s, t, a, b; *Rspo2*: i, j), but they were found in the cytoplasm of oogonia and oocytes after 30dah (*Rspo1*: c, d; *Rspo2*: k, l). *Rspo3* was faintly expressed in the cytoplasm of oogonia and oocytes of adult female gonads (q). No expression of *Rspo1* (e-h), *2* (m-p) and *3* (r) were detected in the male gonads by *ISH* analysis. (▸ = positive signal in the ovary; ⊳ = position of testis; Oo: oogonia; Oc: oocyte; Po: primary oocyte; CA: cortical-alveolar).

*ISH* analysis revealed that three *Rspo* genes were abundant in the ovary, but barely detectable in the testis. Single-color *ISH* analysis showed that both *Rspo1* and *2* were predominantly expressed in the germ cells and germ cell surrounding cells at S38 and 0dah (Figure 5). Later on, their expressions were restricted to the cytoplasm of oogonia, oocytes, primary oocytes and cortical-alveolar stage oocytes from 30dah to adulthood. However, they were not found in late cortical-alveolar stage oocytes, vitellogenic stage oocytes, or the follicular cell layer. The expression of *Rspo3* in XX female gonads could be detected in the adult stage (Figure 5), but was barely detectable during the early ontogenic stages by *ISH* (data not shown).

**Figure 6:**
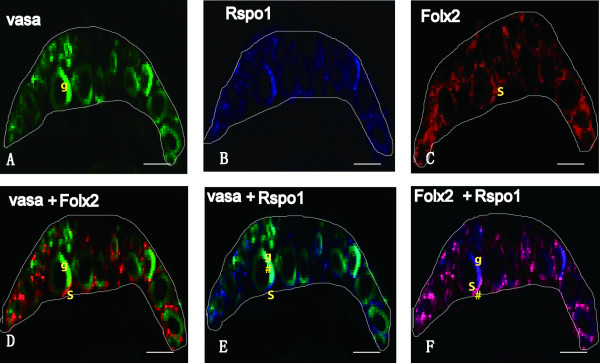
**Localization of *Vasa*, *Rspo1 *and *Foxl2 *in 10dah XX gonads.** RNA probes were labeled with FITC, DIG or Biotin, respectively. Stained sections were observed under a confocal laser microscope. Photographs in A-F show the same area of the XX gonad. The upper panels were the signals of *Vasa* (green) in the germ cells (**A**), *Rspo1* (blue) (**B**) and *Foxl2* (red) in the somatic cells (**C**). The lower panels were the signals of two genes by overlapping. D: *Vasa* and *Foxl2*; E: *Vasa* and *Rspo1*; F: *Rspo1* and *Foxl2*. The # in E indicates co-localization of *Vasa* and *Rspo1* in the germ cells. The # in F indicates co-localization of *Foxl2* and *Rspo1* in the somatic cells. g indicates germ cell and S indicates somatic cell. The boundary of gonad is marked by white color lines.

Fluorescence multi-color *ISH* analysis demonstrated that *Rspo1* was expressed both in the germ cell (overlapping with germ cell marker gene, *Vasa*) and somatic cell surrounding the germ cell (overlapping with follicular cell marker gene, *Foxl2*) at 10dah (Figure 6). The expression of fish *Rspo* genes was not detected in the somatic cell by traditional *ISH* method, which might due to their low expression in the somatic cell surrounding the germ cells.

### Effect of steroid treatment on the expression of *Rspo1*, *2* and *3*

**Figure 7:**
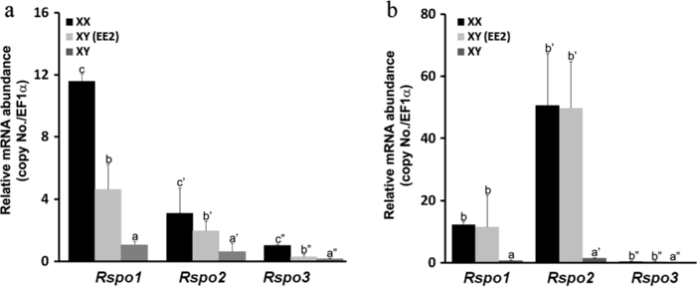
**(a) Changes of the expression profiles of *Rspo1*, *2 *and *3 *after EE2 treatment at 0dah (n = 15).** (**b**) Effect of EE2 treatment on *Rspo1*, *2* and *3* expression in adult XY fish (n = 6). Values (mean ± S.E.) represent the relatively mRNA abundance of *Rspo1*, *2* and *3*. Values with different letters indicate significant difference (P < 0.05) of each gene. (XX, control; XY-EE2, treated group; XY, control).

At 0dah, treatment with EE2 significantly increased the expression of *Rspo1*, *2* and *3* in XY embryos, but levels were much lower than their expression level in control XX embryos (Figure 7a). Moreover, treatment of adult XY fish with EE2 caused significant enhancement of *Rspo1*, *2* and *3* expressions comparing with normal XX female levels (Figure 7b). *ISH* revealed that *Rspo1*, *2* and *3* were up-regulated in the EE2 treated XY gonad at S37, 0dah and adult stage (Additional file 1: Figure S1).

## Discussion

Three members of the Rspo family were cloned and characterized from a teleost fish, medaka. Interestingly, medaka *Rspo1*, *2* and *3* showed a sexually dimorphic expression profile with female-specific up-regulation during the critical period of sex determination and differentiation and later developmental stage. Thus, the abundant expression of these three *Rspo* genes in the female gonad indicated that Rspo-activating pathway might be required for ovarian differentiation and maintenance in fish.

In vertebrates, *Rspo1* displayed a female-specific increase in the gonads of humans, mice, goats, chickens and reptiles during the critical period of sex determination/differentiation [5,17-19]. To date, the expression of *Rspo1, 2* and *3* had merely been identified in goats. It has been reported that goat *Rspo2* was expressed with a female-specific profile from the crucial stage of sex determination until adulthood. However, goat *Rspo3* was expressed equally in females and males [5]. In medaka, it has been shown that sex determination occurred around stage 38 before hatching [29]. At this stage, other than the germ cell markers in females, as well as *DMY* and *GSDF* in males, all known genes have been shown to be expressed without any sexual dimorphism at this stage [29-31]. Our real-time PCR results demonstrated that medaka *Rspo1*, *2* and *3* expression was specifically up-regulated from S38 along with the expression of a germ cell marker (*Vasa*) and meiosis marker (*Spo11*) in the female gonads. A previous report demonstrated that RSPO1/β-catenin signaling is involved in meiosis in fetal germ cells and contributes to the cellular decision of germ cells to differentiate into oocytes or sperms [21]. The expression profiles of medaka *Rspo1*, *2* and *3* during the early sex determination/differentiation stages imply that Rspo signaling might be also required to initiate meiosis in the germ cells of medaka. Therefore, the female-specific expression of the three *Rspo* genes in medaka suggests that they lie upstream in the genetic cascade of female sex determination and differentiation in teleosts.

The sub-cellular distribution of Rspo1 protein during the critical period of sex determination has been well investigated in mice, goats and chickens. In the E14.5 female mouse gonad, the protein was mainly located in germ cells and somatic cells. In the E8.5 chicken ovary, it was widely expressed in the cytoplasm and on the cell surface in the outer cortical zone including both germ and somatic cells, just prior to the onset of meiosis [19]. In goat, the protein was detected around both somatic and germinal cells in the cortical area as early as 36-40*dpc*. At 50*dpc*, the strongest *Rspo1*-specific signal was found around the germ cells in goats [5]. Our multi-color *ISH* data revealed that medaka *Rspo1* mRNA was expressed in both somatic cells (overlapping with *Foxl2*) and germ cells (overlapping with *Vasa*) in the early stages of sex determination (10dah). However, strong expression of *Rspo1* was only detected in the cytoplasm of oocytes at a much later stage. Additionally, up to now, no reports are available on the cellular distribution of *Rspo2* and *3* in vertebrates. In this study, both *Rspo2* and *Rspo3* showed the same expression profiles as *Rspo1* in the gonads during different ontogenic stages in the *ISH* analysis. Concisely, the female-specific expression profile in the somatic (pre-follicular) cells and germ cells during early sexual differentiation suggests a possible role of fish *Rspo* proteins in both folliculogenesis and development of germ cells.

The role of germ cells in sex determination and differentiation differs between mammals and fish. In mammalian species, the presence of PGC doesn’t play an important role in somatic sexual differentiation. Unidirectional signaling from the soma to germ cells has been found to be important for sex determination and differentiation [32]. In fish, previous reports showed that gonadal somatic cells are predisposed to male development in a cell-autonomous fashion. Therefore, in medaka, germ-cell-deficient adults displayed a female-to-male secondary sex reversal phenotype [33]. Similarly, the ablation of germ cells resulted in the generation of sterile males, indicating that the germ cell line is essential for the development of female zebrafish [34]. Therefore, germ cells are necessary for sexual dimorphism and sex differentiation in fish. In this study, the presence of *Rspo* protein in both somatic and germ cells in the developing ovary indicated its essential role in fish sexuality.

Estrogens play a pivotal role in ovarian differentiation and maintenance in non-eutherian vertebrates. Recent reports revealed that Foxl2 plays a decisive role in ovarian differentiation by regulating aromatase expression and possibly the entire estrogen pathway in teleosts [26]. *Foxl2* and *RSPO1* double knockout resulted in sex reversal in XX mice [35]. In chicken, treatment with an aromatase inhibitor (Fadrozole) reduced the expression of *Rspo1*. In the present study, the expression profiles of *Rspo1-3* in gonads were remarkably enhanced during a short period of exposure to estrogen, in both 0dah and adult XY medaka. This result further strengthens the idea that estrogen could induce the activation of Rspo signaling pathway which is required for ovarian development in XY sex reversal females treated with EE2. Evidence in mice and humans suggests that the canonical Wnt signaling pathway promotes ovarian fate and blocks testis development. Duplication of the distal portion of chromosome 1p, which includes both WNT4 and RSPO1, overrides the male program and causes male-to-female sex reversal in XY patients. Ectopical expression of β-catenin in the somatic cells of XY gonads disrupts the male program and results in male-to-female sex-reversal [36,37]. Our preliminary studies showed that over-expression of *Rspo1* in XY medaka disrupts male development, and induces ovarian development (data not shown). Therefore, we hypothesized that like in mammals, the proper development of ovaries requires the interaction and complement of Rspo-activating signaling pathway and Foxl2-leading estrogen producing pathway. However, further investigation is required to test this presumption.

## Conclusions

These results suggest that the Rspo-activating signaling pathway is involved in the ovarian differentiation and maintenance in medaka. Our data also support that estrogen producing pathway and Rspo-activating signaling pathway might be complementary in female sex determination/differentiation in fish.

## Methods

### Fish strains and husbandry

The QurtE strain of medaka (*Oryzias latipes*) was used for gene cloning and expression analysis. All fish were maintained under a 14-h light, 10-h dark photoperiod prior to use. Both Y chromosome-derived lucorphore and genomic PCR (*DMY*-F, *DMY*-R) were used to identify the genetic sex of fish before experiments. All *in vivo* experiments and fish maintenance were conducted following protocols and procedures approved by Institutional Animal care and use committee at the National Institute for Basic Biology, Japan.

### Molecular cloning

A 540 bp fragment encoding *Rspo1* was isolated from the medaka ovary by gene specific primers (1-F1, 1-R1) designing according to the EST sequences of medaka (K05119-53_A06). Subsequently four gene-specific primers were designed to amplify the 5’- and 3’-end cDNA sequence by SMART 5'-rapid amplication of cDNA ends (RACE) and 3'-RACE (1-F2, 1-F3, 1-R2, 1-R3) according to the manufacturer’s instructions. The open reading frame (ORF) of *Rspo2* was obtained from the medaka ovary by gene specific primers (2-F, 2-R) based on the available database (ENSORLT00000025646). A partial sequence of *Rspo3* was amplified (3-F1, 3-R1) in the medaka ovary basing on the available sequence from medaka genome databases (ENSORLT00000007234). The full ORF of *Rspo3* was obtained by RACE (3-F2, 3-R2). All PCR products were ligated into the pGEM-T easy vector (Promega, Madison, WI) and sequenced using an ABI Prism 3100 sequencer (Applied Biosciences, Branchburg, NJ).

### Phylogenetic analysis

The deduced amino acid sequences of medaka Rspo1, 2 and 3 and their counterparts in other vertebrates, as well as Rspo4 from mammalian species were aligned using Clustal W. A phylogenetic tree was generated with PHYLIP software by the neighbor-joining method [38] using mouse-thromobosponding 1 (NP_035710) as an out-group. Values on the tree represent the bootstrap scores of 1000 trials, indicating the credibility of each branch. The GenBank accession nos. of sequences used in this study are as follows, human-RSPO1(NP_001033722), chicken-Rspo1 (XP_417760), *Xenopus*-Rspo1 (NP_001121500.1), zebrafish-Rspo1 (NP_001002352), tilapia-Rspo1 (JF276456), medaka-Rspo1 (JF263584), fugu-Rspo1 (CAAB02002595), human-RSPO2 (XP_001134914), chicken-Rspo2 (XP_418383), *Xenopus*-Rspo2 (NP_001088999), zebrafish-Rspo2 (XP_001919458), medaka-Rspo2 (JF263585), tilapia2 (XP_003453515.1), human-RSPO3 (NP_116173), *Xenopus*-Rspo3 (AAV31038), zebrafish-Rspo3 (NP_001017358), chicken-Rspo3 (XP_419752), tetraodon-Rspo3 (CAG12893), medaka-Rspo3 (JF263586), tilapia3 (XP_003443788.1), human-RSPO4 (EAX10652), mouse-Rspo4 (EDL05937), rat-Rspo4 (XP_575261), chicken-Rspo4 (BAL43044).

### Tissue distribution

For the tissue distribution analysis, total RNA was extracted from brain, heart, liver, ovary, testis, kidney and intestine of adult medaka, according to the manufacturer’s instructions (RNeasy Mini kit, QIAGEN) with RNase-free DNase treatment. Subsequently, reverse transcription for cDNA was conducted (Omniscript RT kit, QIAGEN), and quantitative RT-PCR was carried out to check the levels of *Rspo1*, *2* and *3* in various tissues. The data were analyzed using one-way ANOVA and the least significant difference on the GraphPad Prism 5 software (San Diego, CA, USA).

### Preparation of samples for *ISH*

Whole body specimens of both XX and XY medaka fry at different developmental stages were fixed in 4% paraformaldehyde (Nacalai tesque, Kyoto, Japan) in 0.85x PBS at 4°C as described previously [39]. Probes of sense and antisense digoxigenin (DIG) labeled RNA strands were transcribed *in vitro* with a RNA labeling kit (Roche Diagnostics GmbH, Mannheim, Germany) from plasmid DNA containing the ORF of *Rspo1*, *2* and *3*.

To detect the cellular localization of *Rspo1* during early embryogenesis, fluorescence multi-color *ISH* of *Rspo1*, *Vasa* and *Foxl2* was performed as described previously [40]. Briefly, probes were labeled with fluorescein isothiocyanate (FITC), or DIG or Biotin (Roche, Germany). Horseradish peroxidase-conjugated anti-FITC, anti-DIG and anti-biotin antibodies were used for the detection, respectively. For detection of the signals, a TSA Plus Fluorescein/TMR system was used (Inc., Waltham, MA). Signals were observed and photographed by confocal microscope (Zeiss 710, Carl-Zeiss Germany).

### Real-time PCR

For ontogenic expression analysis of three *Rspo* genes in the medaka gonads, triplicates of five beheaded embryos were collected from both female and male at stage (S) 33, S37, S38, S39, 0dah and 5dah. Subsequent total RNA extraction, cDNA synthesis and real-time PCR were carried out to check the expression of *Rspo1* (1-F4, 1-R4), *2* (2-F2, 2-R2) and *3* (3-F4, 3-R2) as described previously [41]. Data were expressed as the mean ± SE for the 3 replicates. A Kruskal-Wallis test was used to determine significant difference (P < 0.05) with GraphPad Prism 5 software (GraphPad Software, San Diego, CA).

### Treatment with steroid

Vast investigations have proved that exposure to estrogenic chemicals, including natural and synthetic estrogens caused feminization responses or complete sex reversal in male fish. A synthetic estrogen, ethinylestradiol (EE2) is an effective estrogenic chemicals could cause the feminization or sex reversal in vertebrates including fish [42,43].

The effect of EE2 on the expression of the three *Rspo* genes was evaluated by short term immersion of either fertilized eggs or XY adults in aerated fresh water with or without EE2 (10 ng/L) dissolved in ethanol (Wako Pure Chemicals, Japan). The fertilized eggs were treated by EE2 until hatching as described previously and XY embryos carrying lucorphore were sampled at 8dpf (0dah) [44]. RNA extraction and cDNA synthesis were obtained from 3 samples and 5 embryos without head for each sample. For adult XY fish, six male medaka were randomly selected and maintained in aerated fresh water with EE2 (10 ng/L) for one week. The gonads of EE2 treated adult fish were dissected separately for RNA extraction and subsequent cDNA synthesis. Simultaneously, fertilized eggs or adult XY male fishes were assigned to immersion in the same amount of vehicle ethanol in both experiments as control groups, and then RNA extraction and cDNA synthesis also were prepared according to the methods in EE2 treated group. Finally, real-time PCR was carried out to investigate the expression profiles of *Rspo1*, *2* and *3* according to the methods aforementioned. Results are presented as the mean ± S.E. of data from triplicates. The data were analyzed using one-way ANOVA and the least significant difference on the GraphPad Prism 5 software (San Diego, CA, USA). Additionally, *ISH* analysis were carried out to further check the expression changes of *Rspo1*, *2* and *3* genes in EE2 treated XY gonads at S37, 0dah and adult stage. In this experiment, EE2 treatment for XY individual at each stage followed the aforementioned protocol.

Primer sequences used for RT-PCR, RACE and real-time PCR are listed in Table 1.

**Table 1:** Primer sequences used in molecular cloning and real-time PCR analysis

**Primer**	**Sequence**	**Purpose**
*Rspo1*-F1	TGGGACTGGTGGCGCTGGCGATG	fragment amplification
*Rspo1*-R1	GCCTTTCTTAAACCCACATGT	
*Rspo2*-F1	ATGCAGTTTCGACTCTTCTC	
*Rspo2*-R1	TCACTGGCTGGAGCGAGCAG	
*Rspo3*-F1	GCCATGCAATTACAAGTCATCTC	
*Rspo3*-R1	CTACTGCACAAGGCTGTGCTCGGGG	
*Rspo1*-F2	CCCAGAAGGGAATAACCGATGCACAC	3’- RACE
*Rspo1*-F3	CACACAGTGGGCGATGCTACGTCAGC	
*Rspo3*-F2	GGCAGACCCTGCCCTTTGACCACAG	
*Rspo3*-F3	GGCAGACCCTGCCCTTTGACCACAG	
*Rspo1*-R2	CGCTCCAAGAAGATAAAGAGCTTGGG	5’- RACE
*Rspo1*-R2	CTCGTTTCTGCCTTCTCGCCTCCC	
*Rspo1*-F4	AAGTGCGTTGTGCCCAAAACACCG	real-time PCR
*Rspo1*-R4	TCCCATCTTTTCCCTCTCGCCCTAGTC	
*Rspo2-*F2	ACCACCCAAGGACACAATCC	
*Rspo2-*R2	GTGCTTCCCTGAACCACCTC	
*Rspo3*-F4	AAGAGGATCGGGATGAAGCA	
*Rspo3*-R2	TCACACTCCGACCTGCACTT	
*EF1-α*-F	CAGCTTCAACGCTCAGGTCAT	
*EF1-α*-R	TGAACTTGCAGGCGATGTGA	
*DMY*-F	CCGGGTGCCCAA GTGCTCCCGCTG	genomic PCR for the genetic sex
*DMY*-R	GATCGTCCCTCCACAGAGAAGAGA	

## Competing interests

The authors declare that there is no financial or other potential conflict of interests.

## Authors’ contributions

All authors participated in the design of this study. LZ cloned and characterized three types of *Rspo* genes. TC performed the multiple color *ISH*. XY and GL carried out the steroid treatment and tissue collection. LW performed the real-time PCR analysis and all the authors analyzed the results. SM, DW and YN drafted the manuscript. All authors read and approved the final manuscript.

## Supplementary Material

Additional file 1 **Figure S1.** Expression of *Rspo1* (**a-c**), *2* (**d-f**) and *3* (**g-i**) in the EE2 treated XY gonads at S37, 0dah and adult stage. The expressions of three genes were greatly up-regulated in XY gonad by EE2 treatment during three stages. The gonadal boundary is marked by black lines. Scale bar, 50 μm. Click here for file
