# Supplementary figures and images for: R-spondins are involved in the ovarian differentiation in a teleost, medaka (Oryzias latipes)
Source: BMC Dev Biol. 2012 Dec 7;12:36. doi: 10.1186/1471-213X-12-36 (PMC3542121; doi:10.1186/1471-213X-12-36)

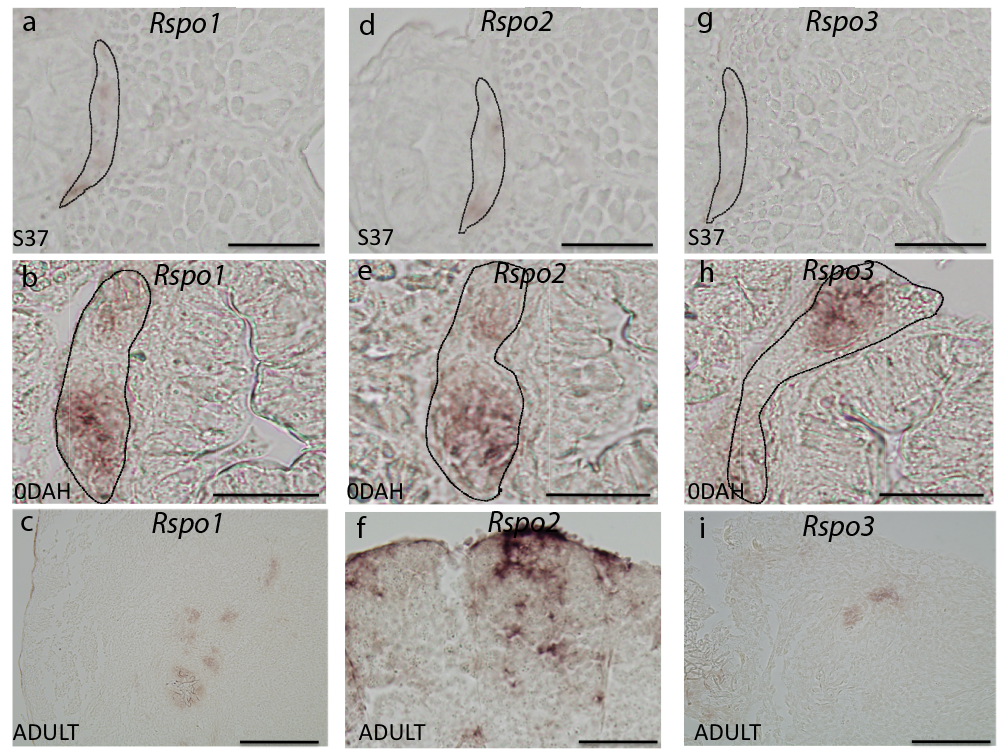

Supplement: Additional file 1 — Figure S1. Expression of Rspo1 (a-c), 2 (d-f) and 3 (g-i) in the EE2 treated XY gonads at S37, 0dah and adult stage. The expressions of three genes were greatly up-regulated in XY gonad by EE2 treatment during three stages. The gonadal boundary is marked by black lines. Scale bar, 50 μm. [file 1471-213X-12-36-S1.tiff]
